# Insulin Resistance: An Unresolved Riddle

**DOI:** 10.3390/jcm12196394

**Published:** 2023-10-07

**Authors:** Patrizio Tatti, Pavandeep Singh

**Affiliations:** Endocrinology and Metabolism Unit, Istituto INI—Grottaferrata, 00046 Rome, Italy

Insulin resistance (IR) is a rather common condition that is often diagnosed on the basis of an arbitrary “increased insulin value” or the presence of symptoms indicative of the Metabolic Syndrome. However, this topic is complex and multifaceted both from a clinical and pathophysiological viewpoint. The definition of Insulin Resistance is useful from a clinical standpoint but could become obsolete over the next few years with the progression of science and a more accurate knowledge of the cellular mechanisms of insulin action. This short review aims to highlight the complexity of the IR and emphasize some unanswered questions.

The hormone insulin is coded on the short arm of Chromosome 11 [1] and synthesized in the β cells of the pancreas as proinsulin. After sequential steps, it is secreted as insulin plus a C-peptide in equimolar ratio [2]. Insulin acts on cells to regulate the metabolism of carbohydrates, lipids, and protein. Insulin is released by the β cells at a rate of 0.25–1.5 U/h [3] in a coordinated fashion, with an ultradian oscillatory pattern [4]. The hormone is also released in a biphasic mode after a meal challenge, with a first rapid peak of greater amplitude, and a more prolonged phase with a lower amplitude to accommodate the differential rate of and absorption of food [5] The term Insulin Resistance refers to an impaired response to the effects of insulin, on glucose disposal [6].

Insulin resistance is a very well-known term that has been equated initially to type 2 diabetes, and in subsequent years, under the name of “syndrome X” or “metabolic syndrome” has been deemed the culprit of a progressive list of chronic diseases, ranging from Impaired Glucose Tolerance and Diabetes to Hypertension, Cancer and Non-Alcoholic Steatohepatitis (NASH) eventually progressing to cirrhosis [7].

In our opinion, the term “Insulin Resistance” (I.R.) tends to obscure the fact that the response of tissues to insulin (better defined as “Insulin Sensitivity, IS”) is a critical regulator of the physiological response of the body to an important hormone. An example of this is the state of pregnancy. During pregnancy, insulin sensitivity decreases (Insulin Resistance increases), mostly in the last trimester, and this represents an adaptive response that improves the delivery of metabolic fuel to the fetus [8]. In other words, the modulation of insulin sensitivity at the tissue level is a prerequisite for survival and is part of a highly regulated system. A reduction in circulating insulin tends to increase sensitivity at the tissue level [9]. Too much insulin sensitivity is as dangerous as Insulin Resistance. We know that the response to the same level of insulin varies in different tissues, and consequently, what can be considered IR at the level of one organ could be absolutely normal in another.

Our present methods of measuring insulin sensitivity allow just a rough evaluationmeasurement of the overall response of all organs to insulin and rely on just one substance: glucose. Only in experimental, expensive, and time-requiring conditions is it possible to locate the IR state in one specific organ or tissue [10]. When there is a gross derangement of this regulatory function, many adverse events appear. However it is quite possible that IR may be localized at the level of just one organ, and this could be the only one affected.

Another aspect of this function and its derangements is that IR might occur at the level of glucose transport into the cell and at subsequent stages of intracellular glucose metabolism, and these may also be involved in IR. Glucose entry into the cell and its metabolism is not the only metabolic pathway affected because insulin also mediates the metabolism of lipids and proteins, and just one pathway could be affected and not simultaneously with the others

There are many hypotheses locating the main cause of the derangement of the IR/IS control system at the level of many organs, notably adipose tissue, but beyond any doubt, there is a prominent role of genetics.

We also know that insulin secretion is pulsatile within a nearly 4 min period, but we do not know if and when the disruption of this rhythm causes what we know as IR. Furthermore, a “pulse” has an amplitude, width, rise and fall time, and many other characteristics that can influence its response at the cellular level. Although there is no consistent proof, it is unlikely that the insulin secretion is independently regulated at the level of the single cell. Rather, it is quite possible that there is a “Pacemaker” (PMK), but the site and the mode of action are still unknown. Dysfunction of this PMK might be one of the culprits of IR, and its discovery could offer a pathway to treatment.

Under normal physiologic conditions, insulin is secreted by the β cells of the pancreas in relation to the prevailing blood sugar levels, possibly under the control of a PMK. Once in circulation, the hormone binds to the peripheral cells of the body. This process is dependent on the number and the type of insulin receptors present on the surface of the cells, which, in turn, are regulated by a number of intracellular factors not completely known. All these activities represent another point of regulation of insulin action far beyond its secretion by the β cell. After binding to the insulin molecule, the receptor undergoes autophosphorilation and sets the path to a cascade of events, which lead to the translocation of the glucose receptor (GLUT-4) to the membrane [11,12,13,14]. Glut-4 is the transporter protein into muscle cells, fat cells, and the heart and has a high Km for glucose transport, thus acting with high selectivity. Glucose uptake into muscle represents roughly 65% of the whole-body glucose uptake mediated by insulin [15]. On the contrary, tissues like the brain, red blood cells, the placenta, and the kidney express GLUT-1 with a much lower affinity for glucose, thus enabling these critical tissues to obtain glucose even in the absence of insulin [16]. This is another example of a highly regulated system. It is, however, worth mentioning that some insulin receptors with high affinity have been located in some areas of the brain and might be involved in appetite regulation and obesity, together with leptin and other peptides [17].

Insulin also has many different actions at the post-membrane level [18]; thus, after the activation of GLUT-4, both glucose and other metabolic pathways involving lipids and proteins are involved [19,20].

A detailed description of the metabolic pathways regulating lipid and protein metabolism through insulin is beyond the scope of this review. However, it is important to remember that insulin works to store lipids in fat cells and suppresses lipolysis. As a consequence, FFAs in the bloodstream are reduced. Insulin also has a permissive role in protein synthesis [21,22] and, even at lower doses, can reduce proteolysis.

The term metabolic syndrome encompasses a wide range of clinical and biochemical manifestations that depend on the impaired action of insulin on tissues. Many clinical and biochemical alterations have been included in the definition of this syndrome throughout the years, but the insulin-resistant state remains at the core.

The WHO defines metabolic syndrome as a pathologic condition characterized by abdominal obesity, insulin resistance, hypertension, and hyperlipidemia. The prevalence of this syndrome is defined by ATP III (≥3 of the following abnormalities): waist circumference greater than 102 cm in men and 88 cm in women; a serum triglyceride level of at least 150 mg/dL (1.69 mmol/L); a high-density lipoprotein cholesterol level of less than 40 mg/dL (1.04 mmol/L) in men and 50 mg/dL (1.29 mmol/L) in women; blood pressure of at least 130/85 mm Hg; or serum glucose level of at least 110 mg/dL (6.1 mmol/L). The syndrome has a prevalence of 20.8 to 42%, according to age in the USA [23], with 22.3% in men and 27.2% in women in Italy [24]. A frequent and dangerous complication of MS is cardiovascular disease (CVD). Part of the explanation for the high prevalence of CVD is that among the many alterations of MS there is the highly atherogenic triad of hypertriglyceridemia, low HDL cholesterol, and the presence of small dense LD particlesL. The most likely mechanism of dyslipidemia in IR states is the missing suppression of lipolysis in adipocytes and the consequent delivery of free fatty acids (FFA) to the liver, which assembles LDL and gives rise to hypertriglyceridemia in the circulation. Under physiologic conditions of normal IS, the degradation of pro atherogenic Apolipoprotein B is increased, while if IS is decreased (IR increased), the degradation of this APO B is impaired. In this condition, the Cholesterol ester transfer protein (CETP) moves Triglycerides to LDL and HDL, and the end result is the presence of small dense LDL and triglyceride-rich HDL that are rapidly cleared from the kidney, thus leaving fewer HDL particles to remove ectopic vascular lipid accumulation [25]. The increased vascular risk during a high IR state is also attributable to the coexistence of hyperglycemia, which causes oxidative stress, endothelial damage, and lipotoxicity caused by the infiltration of lipids in the cardiomyocyte [26].

One recent adjunct to the composite picture of the metabolic syndrome is Non-Alcoholic Fatty Liver Disease (NAFLD) which may progress to the more dangerous Non alcoholic Steatohepatitis (NASH). The pathogenesis of the liver infiltration is essentially the same as in cardiomyocytes. The increased delivery of FFA overloads the liver and causes the ominous consequences described above for the heart. The IR state is also among the causes of Polycystic Ovary Syndrome and menstrual irregularities through the action on the production and conversion of sex hormones.

Although an evaluation of the different methods to calculate IR is beyond the scope of this brief review, it is worth mentioning that there are numerous mathematical models that are easy, cheap, and very useful for evaluating insulin sensitivity. Irrespective of the model used, the greatest value of these techniques is in the repeated measurements. An isolated value, unless is a huge outlier with respect to the reference interval, has limited significance. Because of the need to repeat these measurements, simple methods are preferable in clinical practice.

Among these methods the gold standard is the hyperinsulinemic-euglycemic clamp [27], which is highly requiring in terms of cost and staffing. The continuous infusion of glucose with the model assessment (CIGMA) [28] is simpler and probably under evaluated. Other indexes worth mentioning are the Minimal Model, which can also be used in a shortened version [29], the HOMA2 [30] model, the Insulin Sensitivity Check Index (QUICKY) [31], and the Matsuda Index [32] based on an OGTT.

The objective of this brief review is to underline the complexity of the regulatory system IR/IS, which is also modulated by many hormones and autacoids. We think that the focus should be moved from the interpretation of IR as a pathologic condition to the concept of the derangement of a highly sophisticated system of balance that impacts the physiology and the metabolic well-being of the body. A more detailed comprehension of the mechanisms at the base of this regulatory mechanism is crucial to produce further progress in metabolic disorders. We hope that this Special Issue helps shed some much-needed light on this phenomenon.
